# Multi‐Site Transfer Classification of Major Depressive Disorder: An fMRI Study in 3335 Subjects

**DOI:** 10.1002/advs.202502817

**Published:** 2026-01-29

**Authors:** Jianpo Su, Jian Qin, Hui Shen, Ling‐Li Zeng, Baojuan Li, Jin Liu, Xuemei Song, Li Liu, Weidan Pu, Tongjian Bai, Huaning Wang, Hong Yin, Kai Wang, Lingjiang Li, Dewen Hu

**Affiliations:** ^1^ College of Intelligence Science and Technology National University of Defense Technology Changsha China; ^2^ School of Biomedical Engineering Air Force Medical University Xi'an China; ^3^ and National Clinical Research Center for Mental Disorders The Second Xiangya Hospital of Central South University Changsha China; ^4^ School of Medicine Zhejiang University Hangzhou China; ^5^ College of Electronic Engineering National University of Defense Technology Changsha China; ^6^ Department of Clinical Psychology The Third Xiangya Hospital of Central South University Changsha China; ^7^ Department of Neurology The First Affiliated Hospital of Anhui Medical University Hefei China; ^8^ Department of Psychiatry Xijing Hospital Air Force Medical University Xi'an China; ^9^ Department of Radiology Xijing Hospital Air Force Medical University Xi'an China

**Keywords:** fMRI, graph convolution network, major depressive disorder, multi‐site, transfer learning

## Abstract

Imaging‐based automatic diagnosis of major depressive disorder (MDD) has received widespread attention in precision medicine. Increasing evidence suggests that the pathophysiology of MDD is associated with the abnormality in brain connectome, which could be an effective biomarker for classification. However, previous studies suffered from small number of samples and large multi‐site imaging divergences, as well as irregular graph architectures of the connectome, which challenges the diagnostic classification of MDD. Here, we propose a novel graph convolution network with sparse pooling (GCNSP) to learn the hierarchical features of the connectome graph to improve MDD classification. We applied the model to a multi‐site functional MRI sample (33 sites with 3335 subjects, the largest functional imaging dataset of MDD to date), and perform transfer learning classification for each site using the pre‐trained GCNSP on remaining sites to fit cross‐site divergences, achieving an average accuracy of 70.14%. Moreover, hierarchical dysfunction of default mode network (DMN) is detected by the GCNSP in the patients. The interaction between DMN and frontoparietal network exhibit high discriminative power between patients and controls. Accordingly, this study may provide an effective pipeline for multi‐site diagnostic classification and improve our understanding of hierarchical clues of brain network dysfunction in neuropsychiatric disorders.

## Graphical abstract

The study proposes graph convolution network with sparse pooling to learn the hierarchical features of brain graph for MDD classification. Experiment is done on multi‐site fMRI samples (3335 subjects, the largest functional dataset of MDD to date) and transfer learning is applied, achieving an average accuracy of 70.14%. Hierarchical dysfunction of default mode network is identified in patients.

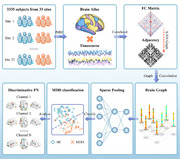

## 1 Introduction

Major depressive disorder (MDD) is among the worldwide leading causes of disability, due to high prevalence, frequently recurrent or chronic character, and its negative impact on the quality of people's life [1]. It was suggested that the general practitioners correctly identified depression only in 47.3% when making routine clinical diagnoses of depression [2]. Therefore, the objective diagnosis classification for MDD has received widespread attention for a long time [2, 3, 4]. Compared with the clinical diagnosis of psychiatric disorders, the neuroimaging‐based diagnostic method has the advantage of being fast and convenient, and not affected by subjective factors [3, 5]. Many studies focus on searching reliable neuroimage biomarkers for diagnosis and treatment of MDD [6, 7, 8, 9]. For example, structural brain abnormalities in patients with MDD are identified based on magnetic resonance imaging (MRI) [10]. As the pathophysiology of MDD was proposed to be associated with the dysfunctional integration of brain regions, the potential of functional connectivity for MDD diagnosis was probed [11, 12, 13, 14]. Zeng, Shen, Liu, Wang, Li, Fang, Zhou, Li and Hu [15] demonstrated that whole‐brain functional connectivity MRI may serve as a sensitive biomarker for identifying patients with MDD from healthy controls. Subsequently, a number of neuroimaging studies further showed the effectiveness of connectome‐based biomarkers in the diagnostic classification of MDD [16, 17, 18, 19].

However, most studies suffered from a small sample and large cross‐site divergences. It is difficult to collect a large number of samples in a single site because of tedious subject recruitment and long scanning time. The neuroimaging data are usually collected at different sites and exhibit huge cross‐site divergences mainly including scanner hardware differences, software differences, and environmental differences [20, 21, 22]. This cross‐site divergences do not only simply affect the mean of the neuroimaging data but also affect the overall distribution of the data, which is difficult to completely eliminate [20, 23, 24]. Therefore, machine learning models that use the same parameters for multiple sites are typically difficult to effectively represent multi‐site neuroimaging data considering the large variability of the cross‐site data distributions [23], such as the models that linear discriminant analysis (RFE‐LDA), logistic regression (RFE‐LR), and support vector machine (RFE‐SVM) combined with recursive feature elimination [25]. Given the small number of single‐site samples, how to effectively represent the multi‐site connectome and then leverage multi‐site data to facilitate pattern analysis on a single‐site remains unsolved.

Transfer learning with the brain connectome may be an effective solution. Transfer learning has been applied for medical image‐based diagnosis and has proven to be effective [26]. We believe that transfer learning may make a model adapt to multi‐sites data and improve its generalization to a single‐site, i.e., we can train the model on large multi‐site datasets and then transfer it to a new single‐site for fine‐tuning to improve performance. Because the brain connectome is lying on the irregular and non‐Euclidean spatial domain, a graph convolution network (GCN), a general convolution technology for graph data [27, 28], was introduced to learn the similarity between different connectome graphs and achieved well performance [29]. GCN considers the adjacency relation of brain regions when learning connectome embeddings, instead of ignoring the spatial information of the connectome as usual [28]. Moreover, GCN required few trainable parameters due to the mechanism of convolutional networks that local connection and weight sharing. Therefore, GCN is more powerful for representing the connectome graph and may be more conducive to multi‐site transfer learning. In general, GCN can directly learn node‐level representation. To classifying MDD patients with the entire connectome graph, it is necessary to effectively aggregate the features of all nodes to form a graph‐level representation.

**FIGURE 1:**
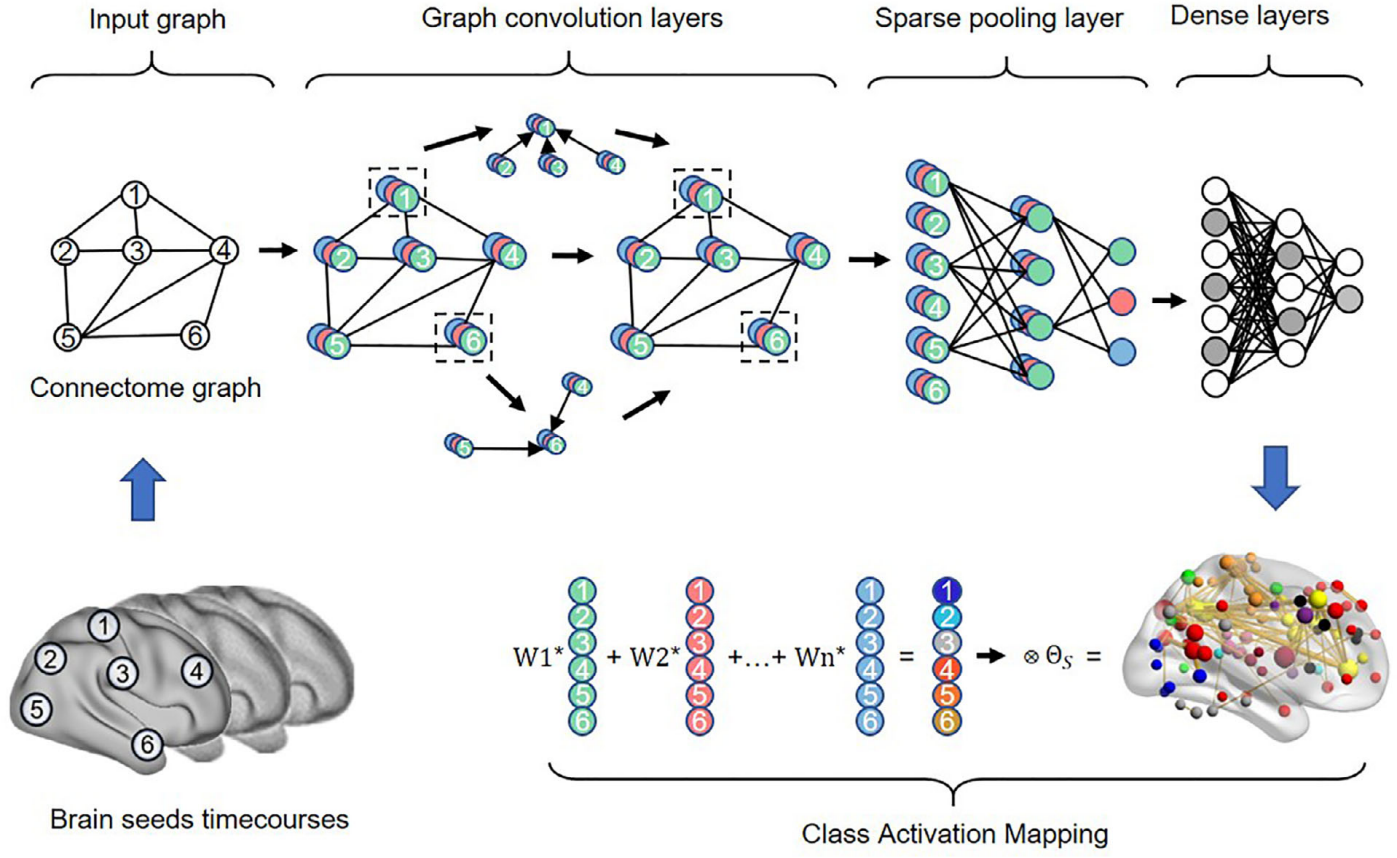
The network architecture of graph convolution with sparse pooling. The node‐based functional connectivity was calculated, and the subject's brain graph was built by taking the ROI as the node, functional connectivity as the edge, and the connections between the current node and all nodes as the features of the current node. Subsequently, the brain graph of each subject, as a sample, passes through the graph convolution layers, the sparse pooling layers, and the dense layers successively. Finally, the two output nodes of dense connection layers could classify the graph label via the softmax function. In addition, the discriminative connectivity to classification can be reconstructed by calculating the class activation map.

**FIGURE 2:**
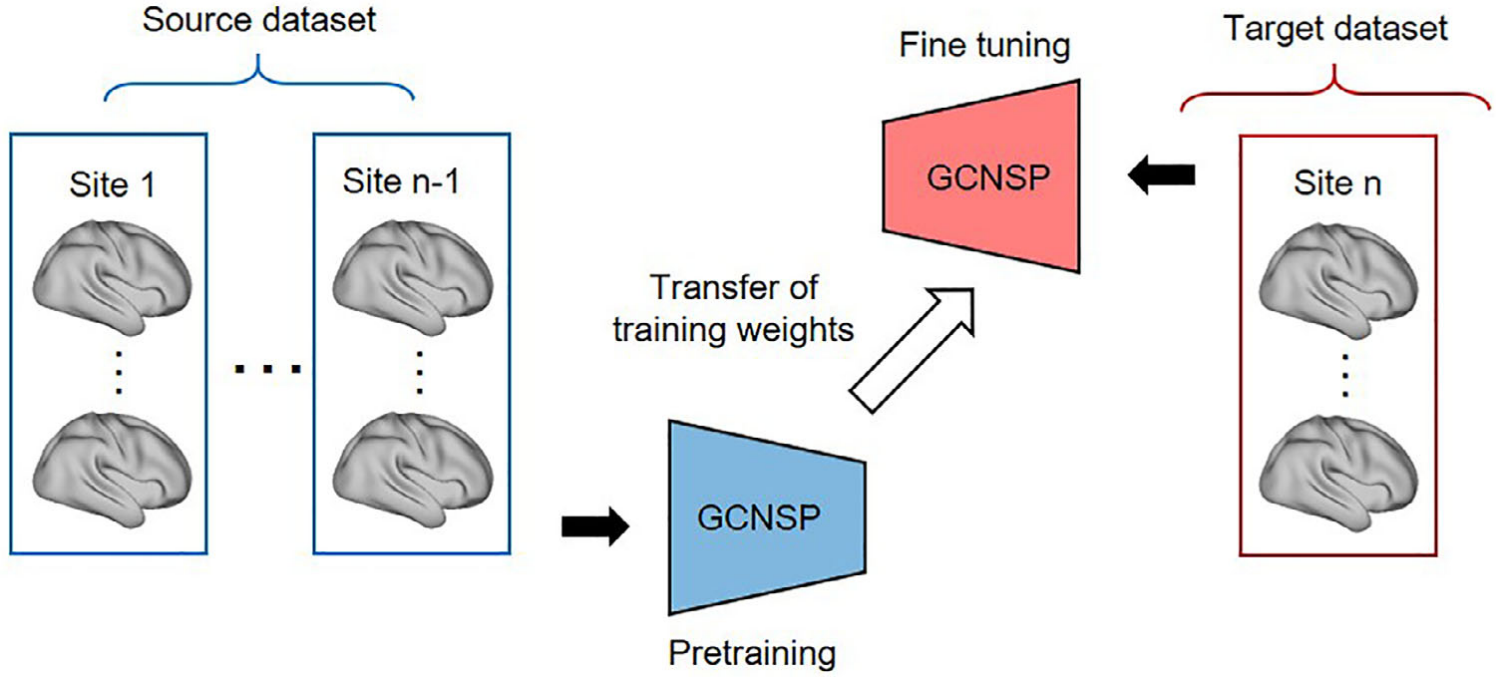
The flowchart of multi‐site transfer learning via GCNSP. The proposed model was pre‐trained in the source dataset and then transferred to the target dataset for fine‐tuning to shorten training time and improve classification accuracy.

Here, we proposed a transferable graph convolution network with sparse pooling (GCNSP) for representing connectome graphs (See Figure 1 for network architecture), which is capable of automatically learning graph‐level hierarchical embedding of connectome graphs. We performed leave‐site‐out transfer learning classification on a large‐scale multi‐site dataset (33 sites, 1768 patients with MDD and 1567 healthy controls; see Table S1 and Figure S1 for subject information), which is the largest MDD sample we know of so far. Specifically, the GCNSP was pre‐trained on multi‐sites and transferred to a new single‐site for fine‐tuning to shorten the training time and improve the classification accuracy (See Figure 2 for flowchart). The results demonstrated that transfer learning with multi‐site connectome graphs using the proposed GCNSP could significantly improve classification performance on single‐site, compared to the baseline of RFE‐SVM or a non‐transfer GCNSP model. In addition, we reconstructed the feature maps of each graph convolution layer which may reflect the hierarchical functional patterns of the human brain, and then investigated the hierarchical dysfunctional networks associated with MDD pathophysiology. Finally, we identified the MDD‐related most discriminative functional networks using the class activation map technique. Our study established an effective framework of transfer learning with multi‐site connectome graphs, which provides new light for the multi‐site transfer learning of psychiatry disorders and advances our understanding of the pathophysiology of MDD.

## 2 Results

### 2.1 Classification with Multi‐Site Connectome Graph

**FIGURE 3:**
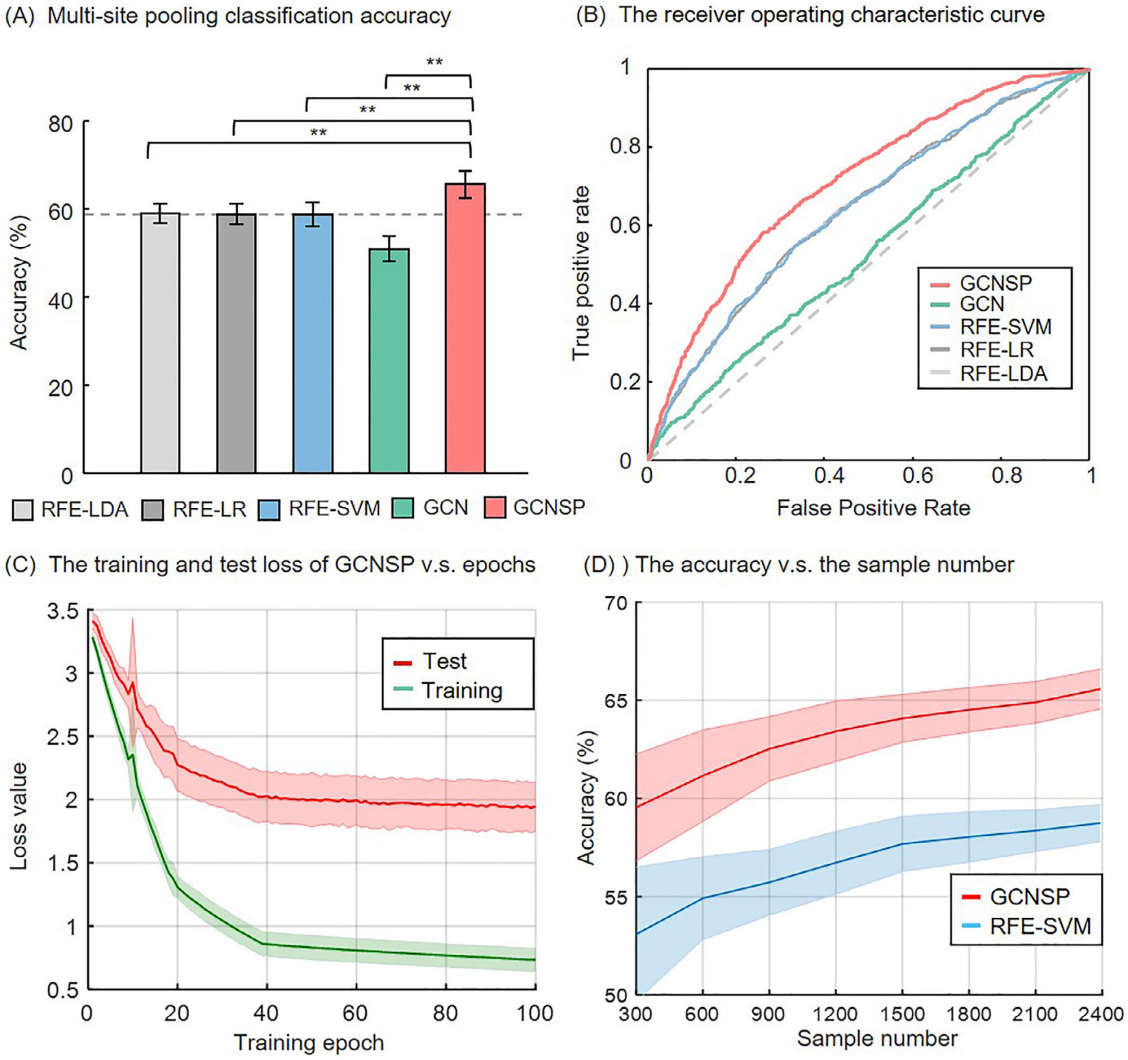
The results of multi‐site pooling classification. (A) The accuracy of ten‐fold multi‐site pooling classification, (B) The receiver operating characteristic curve, (C) The training and test loss of GCNSP changes with training epochs, and (D) the effects of the sample size on classification accuracy. Note that different numbers of samples were randomly selected from the entire datasets by repeating 50 times. RFE, recursive feature elimination; LDA, linear discriminant analysis; LR, logistic regression; SVM, support vector machine; GCN, graph convolution network; GCNSP, graph convolution network with sparse pooling. ^**^ represent *p* < 0.05 in the Kolmogorov‐Smirnov test.

To show the performance of the GCNSP model, we performed the ten‐fold multi‐site pooling classification. As shown in Figure 3A, our GCNSP model achieved the higher accuracy of 65.58% (*p* < 0.05, Kolmogorov‐Smirnov test), compared to other commonly used methods including RFE‐LDA, RFE‐SVM, RFE‐LR, and general GCN classifiers. Figure 3B shows that the receiver operating characteristic (ROC) curve of our GCNSP model also has a larger area under the curve (AUC; 71.21%). The training and test loss of our GCNSP model eventually converge as the training epoch increases (see Figure 3C). Figure 3D shows that the classification accuracy via RFE‐SVM and GCNSP models increases with the number of samples, indicating the importance of training with large‐scale datasets. To show the cross‐site generalizability of the model, we also performed leave‐site‐out test classification, and our GCNSP model achieved the higher accuracy of 62.09% (see Figure S2). Note that the accuracy of leave‐site‐out test classification was lower than that of multi‐site pooling classification, probably because the former did not use the information of the test sites.

**FIGURE 4:**
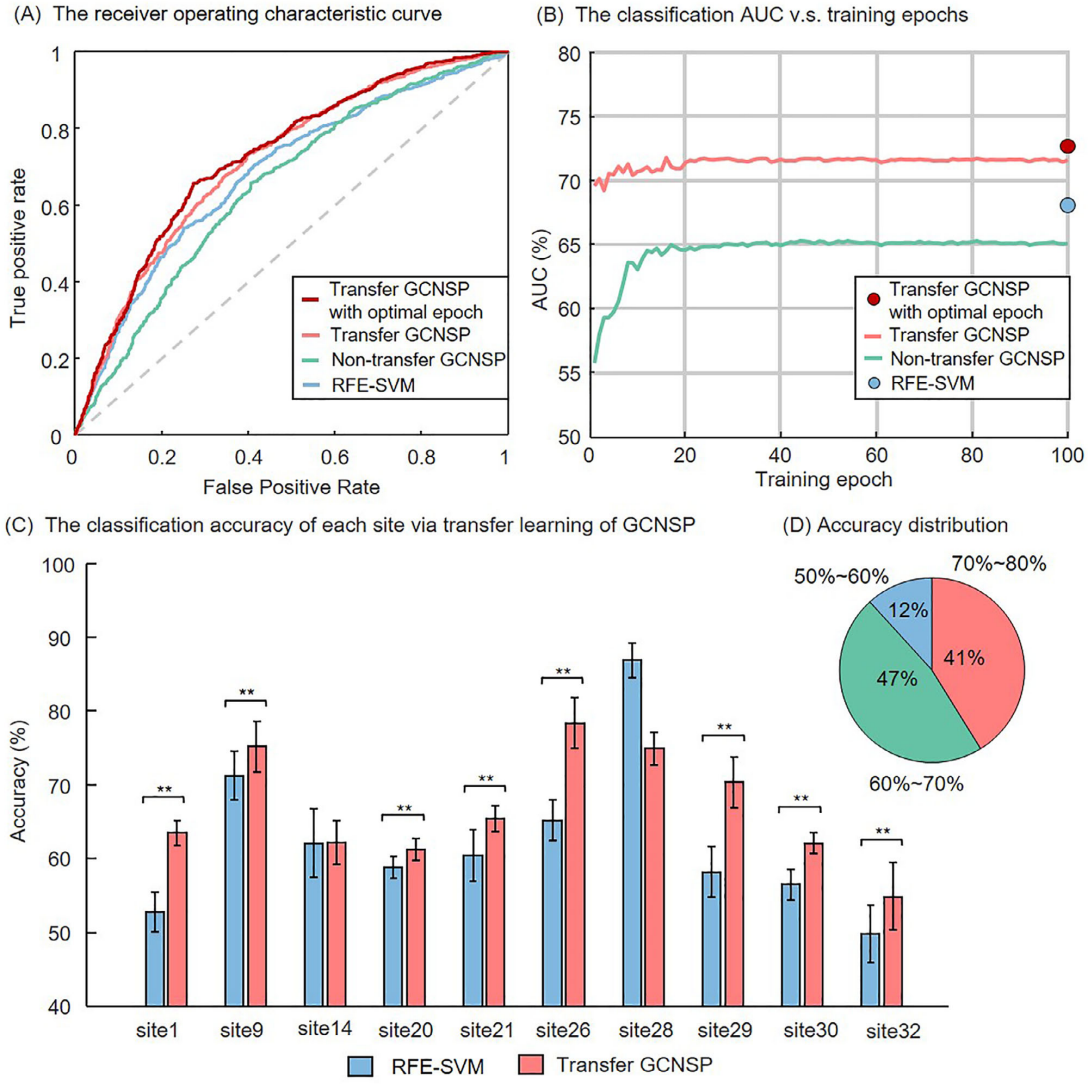
(A) The average receiver operating characteristic curve of RFE‐SVM, non‐transfer GCNSP, and transfer GCNSP on each site. (B) The average classification AUC of multiple models changes with different training epochs. (C) The classification accuracy for each site based on RFE‐SVM and transfer learning of GCNSP. (D) The accuracy distribution of each site based on the transfer learning of GCNSP. Note that the classification accuracy of each site was evaluated based on the ten‐fold cross validation. For transfer learning, a single site was used as the target dataset, and the remaining sites were used as the source dataset, which was denoted as leave‐site‐out transfer learning classification. The transfer learning of GCNSP significantly improved the classification accuracy of each site compared with the baseline of RFE‐SVM. AUC, area under curve; RFE, recursive feature elimination; SVM, support vector machine; GCNSP, graph convolution network with sparse pooling. ^**^ represent *p* < 0.05 in Kolmogorov‐Smirnov test.

In leave‐site‐out transfer learning classification, as shown in Table 1, the average classification results on each site are calculated via the RFE‐SVM, the GCNSP and transfer learning of GCNSP, respectively. Moreover, we also include other studies which also utilize GCN for MDD classification using the REST‐meta‐MDD dataset as a comparison [30, 31]. The average ROC and the ROC on each site of multiple models are shown in Figure 4A and Figure S3, respectively. Specifically, the average AUC of 71.62% (Accuracy of 66.75%) was obtained by using transfer learning of GCNSP, which increases by 6.53% (4.77%) relative to that using non‐transfer GCNSP, and increases by 3.29% (4.50%) relative to that using the RFE‐SVM. Note that the classification based on transfer learning of GCNSP could be further improved to an AUC of 72.77% (Accuracy of 70.14%) by selecting optimal training epoch for each site (see Materials and Methods for more details). The comparison of classification accuracy on each site using RFE‐SVM and transfer learning of GCNSP was shown in Figure 4 (top‐10 sites with the largest samples) and Figure S4 (the selected 17 sites; see Materials and Methods). Note that transfer learning of GCNSP significantly improves the classification accuracy on most sites (*p* < 0.05, Kolmogorov‐Smirnov test).

**TABLE 1:** The average classification results of all sites via multiple models.

Model	AUC (%)	Sensitivity (%)	Specificity (%)	Accuracy (%)
RFE‐SVM	68.33	58.60	61.99	62.25
Non‐transfer GCNSP	65.09	62.81	60.51	61.98
Transfer GCNSP	71.62	71.27	59.44	66.75
Transfer GCNSP with optimal epochs	**72.77**	**73.02**	**66.84**	**70.14**
GCN [31]	∖	∖	∖	63.13
UFA‐Net [30]	62.50	69.46	50.00	59.73

*Note*: “Transfer GCNSP” means fine‐tuning the source model in the target dataset with the pre‐determined number of epochs. “Transfer GCNSP with optimal epochs” corresponds to the highest validation accuracy during fine‐tuning.

In order to assess the performance upper bound of our GCNSP model, we also performed an intriguing gender classification on a large health sample [32, 33, 34]. We performed leave‐site‐out transfer learning classification on a large‐scale multi‐site health dataset (1 8312 fMRI images from 8474 subjects, see Table S2 for more details), and achieved the average AUC of 94.09% (Accuracy of 87.21%) that increases by an average of 4.06% (5.35%) relative to non‐transfer GCNSP for each site (see Figure S5). Furthermore, the AUC exceed 95% on the Human Connectome Project (HCP; AUC = 97.66%, Accuracy = 91.93%) [35], the Brain Genomics Superstruct Project (GSP; AUC = 96.68%, Accuracy = 90.95%) [36], and the Southwest University Longitudinal Imaging Multimodal (SLIM; AUC = 95.79%, Accuracy = 91.73%) [37].

### 2.2 Feature Map of Each Layer of GCNSP Model

**FIGURE 5:**
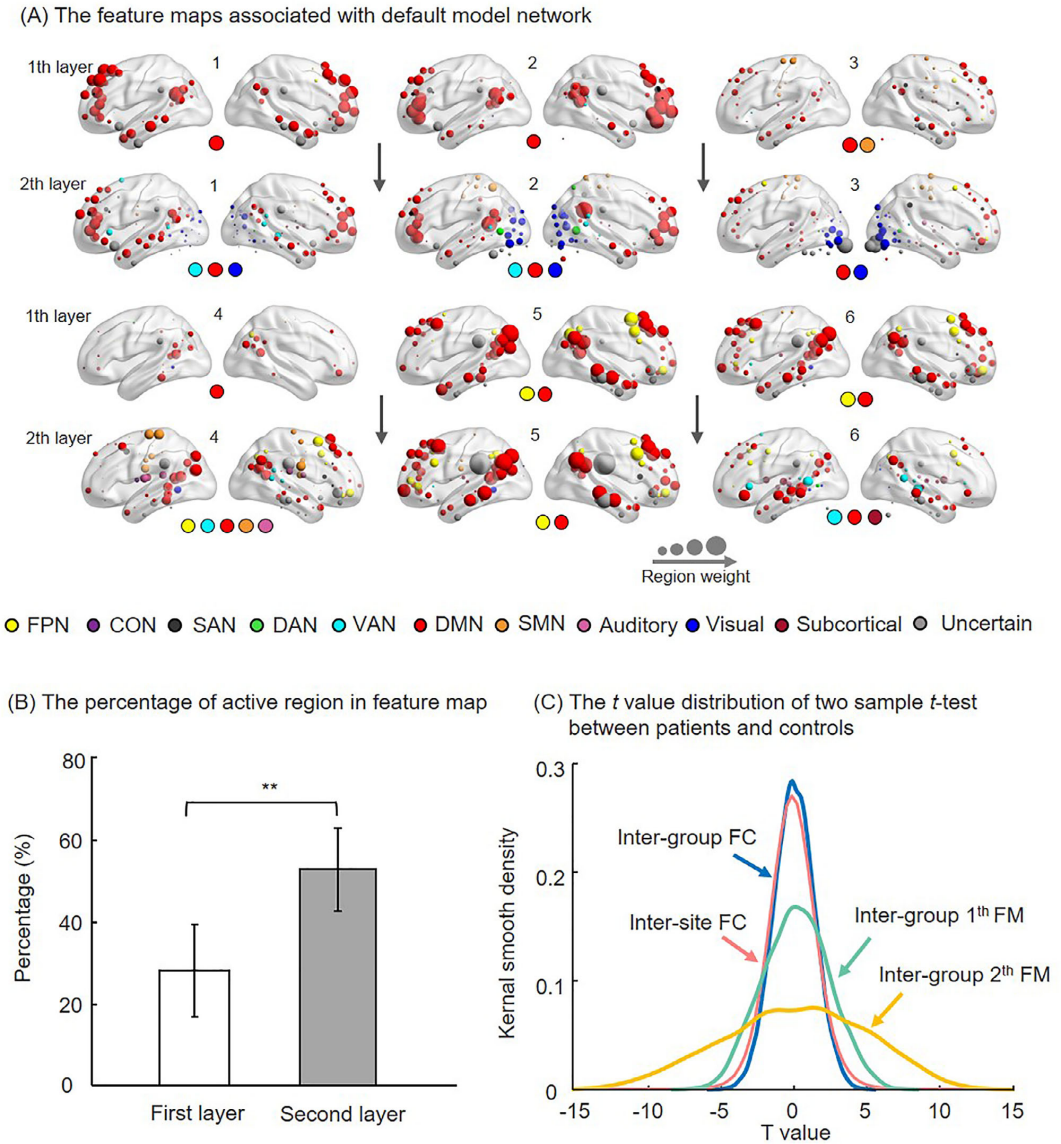
The feature maps of each layer associated with default mode network (A) The sphere represents the region of interest, and the size denotes the weight contributed to classification and the colour denotes the brain functional network. The number at the top of the feature map indicates the channels, and the coloured sphere at the bottom represents the discriminative network. (B) The percentage of discriminative region in feature map of each layer. (C) The distribution of *t* value of two sample *t*‐test on functional connectivity and feature maps between patients and controls (inter‐group), as well as between different imaging sites (inter‐site). ^**^ represent *p* < 0.05 in Kolmogorov‐Smirnov test. FC, functional connectivity; FM, feature map.

To identify the brain activity patterns learned by the GCNSP model, we visualized the average feature map across subjects for each channel for each network layer (see Figure S6). In order to facilitate further analysis, we identified the feature maps associated with the default mode network (DMN), and visualized similar feature maps between the two network layers together (see Figure 5A). We found that different feature maps may reflect different pattern and interaction of brain functional networks. Among them, 12 feature maps (37.5%) are associated with DMN, and the percentage was still significantly higher than other networks even after network size correction. Most importantly, feature maps in the high layer have more discriminative brain regions than those in the lower layer (Figure 5B), which demonstrates that the proposed graph convolutional network can help to reveal the underlying connectivity patterns related to MDD through cascading convolutional layers. The revealed patterns can then improve the classification accuracy of MDD and enhance the understanding of the functional connectivity related to the disorder. Specifically, the first layer captured the abnormality within brain networks, and the second layer captured the abnormality of the interaction between networks. Finally, we found that feature maps of deeper layers show greater differences between patients and controls by performing two sample *t*‐test (Figure 5C). Note that the larger the value at both ends of the distribution probability curve, the greater the difference between the groups (see Materials and Methods for more details).

### 2.3 The Most Discriminative Functional Connectivity

**FIGURE 6:**
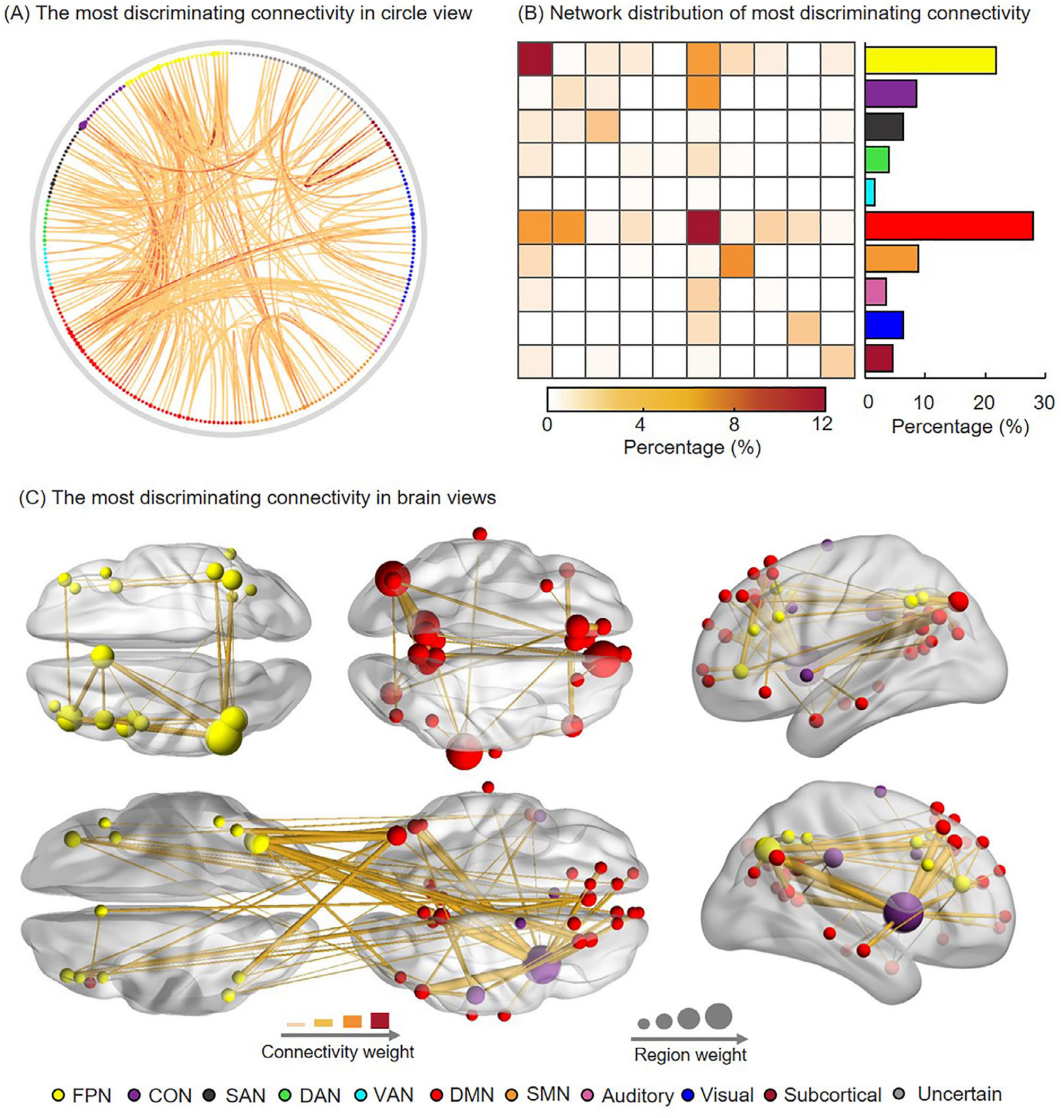
(A) The most discriminative functional connectivity in circle graph. The discriminative functional connectivity was reconstructed via class activation map technology, and only the top 1% of the discriminative connectivity was shown. The connectivity weight represents the contribution to classification. (B) The network distribution of the most discriminative connectivity. (C) The discriminative connectivity related to the default mode network, frontoparietal network, and cingulo‐opercular network.

To evaluate the contribution of different brain connectome to classification, we calculated the class activation map for patients with MDD, which can be considered as a discriminative connectome for classification (see Materials and Methods for more details). The top 1% of the most discriminative connectome with the largest value were shown in Figure 6A. Then we calculated the discriminative network weights by averaging the class activation value of the most discriminative connectome within and between networks (see Figure 6B). The functional connectivity within DMN and the frontoparietal network (FPN) shows the greatest weight. In addition, the connectivity between the DMN and FPN, as well as the connectivity between the DMN and cingulo‐opercular network (CON), both exhibits large weight. According to previous researches, the global FPN connectivity contributes to the regulation of mental health symptoms across both health and MDD patients [38], while the alteration of CON is associated with suicide thoughts and behaviors in MDD patients [39]. These findings could deepen our understanding of the pathogenesis of MDD in brain architecture and brain connectome graphs.

### 2.4 Control Analysis Results

First, we calculated the feature map and discriminative connectome in data from several large single sites and evaluated the consistency of the results. We found that the key results were stable based on different single sites, which can exclude the dominant influence of site bias. Second, we excluded some very messy data from 1.5T scanner and older scanning protocols, and performed the multi‐site pooling classification on the remaining data by using GCNSP. Compared with the whole data, we achieved a higher classification accuracy on the subset. Third, in order to evaluate the potential effect of the hierarchical model, we performed an additional control analysis. We randomly shuffle the adjacency matrix of the brain connectivity graph, and applied the same GCN model for feature map reconstruction. We found that the shuffled data cannot get hierarchical feature maps. We also shuffle the node order of the input brain graph and also found that the shuffled data cannot get hierarchical feature maps. The above results demonstrate that the hierarchical nature of the features is not caused by the hierarchical model.

## 3 Discussion

We have proposed an effective framework that transfer learning of the GCNSP model to learn embeddings of the brain connectome graph and discriminative MDD patients from healthy controls in a large multi‐site sample. Our GCNSP model shows better performance both in multi‐site pooling classification and leave‐site‐out test classification compared with other popular methods. Importantly, the average AUC of 71.62% for each site was obtained by using transfer learning of GCNSP, which increases by 6.53% relative to that using the non‐transfer GCNSP model, and increases by 3.29% relative to that using the RFE‐SVM, suggesting the advantages of transfer learning with GCNSP on connectome graph in diagnosis classification of MDD patients across multiple independent imaging sites. In addition, the feature map of each layer of GCNSP reflects hierarchical activation of brain functional networks and dysfunctional integration at multi‐level in patients with MDD. Finally, the functional connectivity of DMN, FPN and CON shows the largest weight in classification between patients and controls across multiple imaging sites, suggesting the reliable biomarkers and dysconnectivity model underlying the pathophysiology of patients with MDD.

### 3.1 Transfer Learning with GCNSP Improves the Classification Performance of Connectome Graphs

The neuroimaging‐based diagnostic classification of patients with MDD is attracted much attentions. The key issue is to find reliable neuroimaging‐based biomarkers for diagnostic classification. Although a number of studies have probed the potential of connectome‐based biomarkers in discriminative patients with MDD [15, 18, 19], previous studies suffered from small sample size and large cross‐site divergences, which may lead to overfitting, poor cross‐site generalization and site‐specific biases of the classification model. The current study developed an effective framework of transfer learning with the GCNSP model, which significantly improves the classification AUC on each site via leveraging multi‐site connectome graphs (the accuracy increases by 6.53% relative to non‐transfer GCNSP, and increases by 3.29% relative to RFE‐SVM). The promising classification results may derive form the following aspects: First, the proposed GCNSP has a stronger feature extraction ability than traditional machine learning methods for connectome graph with irregular domain, and only has a small number of training parameters because of the mechanism of local connections and parameter sharing [28], which make graph convolution very suitable for the pattern analysis of connectome graphs with small sample size and high‐dimensional features [29]. Second, the proposed GCNSP can learn to select the important sparse nodes for pooling or coarsening the graph, which can extract the hierarchical graph embeddings by automatic learning, thus helping to improve classification performance and transfer capability. Note that in the graph classification task, it is necessary to effectively aggregate the features of all nodes to form a graph‐level representation [40]. Third, the GCNSP may learn the multi‐site invariant patterns and then adapt those patterns to a new site via transfer learning to overcome cross‐site divergences. Note that traditional classification models are usually difficult to work directly across multi‐sites of fMRI data, because the diverse differences between data sites could introduce structured noises, which includes scanner hardware differences (manufacturer, head‐coil), software differences (filters, k‐space acquisition method, scan parameters), and environmental differences (radio‐frequency noise) [20, 21, 23, 41, 42]. Our framework has important implications and applications for improving multi‐site classification of MDD based on connectome graphs. It should be noted that the accuracy of the transfer learning on several sites was slightly lower than the accuracy of the GCNSP (see Figure 4C; Figure S4), which may be due to the large individual differences and too specific data distribution of these sites, resulting in the pre‐trained model not working.

The final average classification AUC based on transfer learning of GCNSP was improved to 72.77% (Accuracy of 70.14%) by selecting the optimal training epoch for each site. In the future, multi‐modal fusion may improve the classification further to achieve the clinically relevant accuracy threshold of 80%, and thereby promote the translation of pattern classification of brain imaging from bench to  bedside. It should be noted that, in order to evaluate the generalization of the GCNSP in large‐scale data, we kept as much data as possible. Given high‐quality data, GCNSP's classification performance can reach the ceiling. For example, using the transfer learning of GCNSP for gender classification, we achieved a state‐of‐the‐art classification AUC exceeds 95% (Accuracy > 90%) on the HCP, GSP, and SLIM datasets.

In addition to multi‐site differences, multiple neurophysiological subtypes are also important issues in the MDD classification. Considering the differences in MDD subtypes, Drysdale, Grosenick, Downar, Dunlop, Mansouri, Meng, Fetcho, Zebley, Oathes and Etkin [18] improved the classification of patients vs. controls by clustering patients into four subtypes and applied different classifiers for different subtypes. In contrast, our model is an end‐to‐end architecture that can be implemented for integrated parameter optimization, as well as for transfer to new sites with pre‐trained models. Moreover, the pre‐trained model is easily updated continuously with new samples, making the model better suited for clinical applications where data are increasing. Collectively, based on different perspectives (subtypes divergences vs. multi‐site divergences), our model and the pipeline proposed by Drysdale, Grosenick, Downar, Dunlop, Mansouri, Meng, Fetcho, Zebley, Oathes and Etkin [18] both provide new inspirations for the diagnostic classification of neuropsychiatric disorders.

The differences in symptom severity, medication status and current mood state will further increase the individual differences, which may hinder the improvement of classification accuracy and the reproducibility of the results. Correlation analysis between site‐specific factors, i.e., sample size, scanner types and patient demographics, have also been done to further unveil the potential source for poor classification performance (Table S3). Results indicate that the age of patients, severity of symptom and magnetic field intensity may be important factors of MDD classification. Patients with older ages, more severe symptom and higher‐resolution brain images may be easier to be identified. Moreover, the heterogeneity in neurovascular coupling may lead to a controversial understanding of fMRI signals. This may be the underlying mechanism of the moderate classification accuracies in the study [43].

There are various ways that may make some contributions to the improvement of classification performance. On the one hand, more advanced machine learning algorithm can help. For example, the fusion of multi‐modal brain images, such as MRI and functional MRI. It is important to design an appropriate model that can fuse the features of different modalities and facilitate classification. Besides, designing classifiers for different MDD sub‐types may also help due to the heterogeneity of the disorder. On the other hand, stricter rules should be applied on the inclusion of subjects as well as image acquisition. For examples, only including first‐episode patients with high HAMD and scanners should have a magnetic field intensity of at least 3 Tesla. By integrating the above approaches, the classification performance may be improved and finally achieve the clinical requirement (typically > 80%).

### 3.2 Hierarchical Activation and Dysfunction of Brain Networks

Previous studies suggested that functional brain networks have different subnets and dynamic network boundaries [44, 45, 46], which indicates that brain networks may include multiple states and different between‐network interactions. How to capture hierarchical activation and dysfunction of brain functional networks is a big challenge. In the present study, through cascading graph convolution layers and fully connected layers, the embedded features in the last layer can incorporate the inherent connectivity architecture of the whole brain. Both localized and long‐range connectivity can then be embedded and help to identify patients with MDD. In this way, either localized abnormalities or widespread collaborative dysfunction can be modelled and identified [47]. Moreover, the complex correspondence between symptoms and functional connectivity can also be modeled and explained using the cascading graph convolution layers according to the global topographic theory [48, 49, 50]. We also identified hierarchical activation patterns of brain functional networks by learning feature maps on different channels of multiple layers. Subsequently, we evaluated hierarchical dysfunction by calculating feature map differences between patients and controls. The results show that the same brain network exhibits different activation patterns, which may reflect the different states of the brain network. For example, the feature maps of the first layer shown in Figure 5A mainly reflect the different activation states of DMN. Moreover, multiple networks could activate simultaneously on a feature map, which may reflect the interaction patterns between brain networks. For example, 2th and 3th feature maps of the second layer mainly includes DMN and visual network, nineth feature map of the second layer mainly reflects the interaction between SMN and visual network (Figure S6). This can be well explained by theory proposed by Georg Northoff, which suggests that the brain‐symptom relationship should be analyzed using a global topographic approach. That is, multiple brain regions or networks can be simultaneously related to one symptom while several symptoms can be related to one brain region or network [48, 49, 50]. Most importantly, by comparing the feature maps of different network layers, we demonstrated that the brain connectome in MDD presents a topographic reorganization with a shift from unimodal to transmodal regions [50]. That is, the deeper the network layer, the more transmodal brain regions (Figure 5B), suggesting that the deeper layer captures the inter‐network activation of brain regions, while the shallow layer captures the unimodal activation [51]. Finally, we found that feature maps of deep layers show greater differences between patients and controls (Figure 5C), suggesting that the pathophysiology of MDD was mainly associated with the dysfunction of transmodal brain regions. According to previous research, the shift from unimodal to transmodal regions in MDD may be caused by dynamic shifts from shorter to longer timescales or abnormalities in the excitation‐inhibition balance [48].

### 3.3 The Most Discriminative Functional Networks

Compared with previous studies, we identified the reliable most discriminative functional networks using a large sample across multiple imaging sites. By reconstructing the class activation map of functional connectivity, we demonstrated that the DMN plays a key role in patients with MDD, which was a commonly recognized brain network in MDD studies [52]. Specifically, the functional connectivity within DMN and FPN shows the greatest weight. Moreover, the connectivity between the DMN and FPN, as well as the connectivity between the DMN and CON, both exhibit large weights. Note that DMN has been shown to be involved in reflections upon self [53], self‐emotional state [54], thinking about others, and planning for the future [55], and FPN plays a privileged role as a flexible hub of cognitive control [56, 57], and CON is associated with maintaining alertness attention [58, 59]. Therefore, the abnormality of DMN, FPN and CON may be the underlying neural pathophysiology of cognitive and emotional disorders in patients with MDD. Most importantly, the discriminative DMN‐FPN connectivity and DMN‐CON connectivity were associated with different brain regions of DMN, which well match the findings in previous research [50]. It is suggested that the within‐network resting‐state functional connectivity alteration of DMN in MDD are caused by the brain's global activity rather than local. These finding further suggest that MDD may be a disorder of the spatial topography of the brain's global activity, as postulated in the “resting state hypothesis of depression” [60].

## 4 Outlook

In the present study, we proposed a novel framework for transfer learning with multi‐site connectome graphs to help significantly improve the classification of MDD for a single site. The proposed GCNSP extends the convolution operation to the brain connectome graph and achieves a powerful performance of transfer learning classification on a large‐scale multi‐site sample. Moreover, we provided the pre‐trained model trained with large samples, which can be applied to improve the classification for new imaging sites, and may promote the study of automatic diagnosis of psychiatric disorders. In addition, we found that the hierarchical dysfunctional DMN was associated with the pathophysiology of MDD and the high‐level activation of the brain network exhibits greater differences between patients and controls. Finally, by reconstructing the most discriminative functional connectivity, we showed that the connectivity within DMN and FPN, the DMN‐FPN connectivity, and the DMN‐CON connectivity exhibit the largest weight for classification. These findings could deepen our understanding of the pathophysiology of MDD.

## 5 Materials and Methods

### 5.1 Participants and Data Acquisition

We applied a large‐scale multi‐site sample, including 1768 patients with MDD and 1567 healthy controls from 33 imaging sites. The collected subjects were matched for gender, age, and education. The study patients were diagnosed as MDD based on ICD10 or DSM‐IV. All participants provided written informed consent at their local institution. Specifically, we collected a dataset includes 468 patients with major depressive disorder and 439 healthy controls from 8 imaging resources, including the First Affiliated Hospital of Anhui Medical University in China (AMU: 106 patients and 47 controls), the First Affiliated Hospital of China Medical University (CMU: 24 patients and 29 controls), the Xijing Hospital in China (Xijing#1: 62 patients and 67 healthy controls with 134 images; Xijing#2: 43 patients and 59 healthy controls), the Second Xiangya Hospital in China (Xiangya#1: 158 patients and 117 controls; Xiangya#2: 20 patients and 43 controls; Xiangya#3: 42 patients and 51 controls), and the Zhejiang University School of Medicine (ZUSM: 13 patients and 26 controls). The remaining samples were obtained from the REST‐meta‐MDD Project (http://rfmri.org/REST‐meta‐MDD) [61], including 1300 patients with MDD and 1128 healthy controls from 25 imaging sites. The 562 patients with first‐episode MDD included 318 first‐episode drug‐naïve (FEDN) MDD and 160 scanned while receiving antidepressants (medication status unavailable for 84). Of 282 with recurrent MDD, 121 were scanned while receiving antidepressants, and 76 were not being treated with medication (medication status unavailable for 85). Episodicity (first or recurrent) and medication status were unavailable for 456 patients. The acquisition parameters of imaging data and subject information for each site were described in (Table S1 and Figure S1). Note that data of site 4 (24 patients and 24 controls) was excluded from the present study, because it duplicated from site 14. Data of site 19 (51 patients and 36 controls) was excluded, because it did not provide the necessary time series for calculating the connectome. 127 subjects (80 patients and 47 controls) with abnormal values of connection strength (outside the interval of [−1, 1]) were excluded. Furthermore, we implemented the same exclusion criteria as Yan, Chen, Li, Castellanos, Bai, Bo, Cao, Chen, Chen and Chen [61] (e.g., incomplete information, bad spatial normalization, bad coverage, excessive head motion greater than 0.2 mm, and sites with fewer than 10 subjects in either group), obtaining 2325 subjects (1200 patients and 1125 controls) from 23 sites for further analysis. More details were described in the Supporting Information.

### 5.2 Data Preprocessing

A standardized preprocessing protocol on Data Processing Assistant for Resting‐State fMRI (DPARSF; http://rfmri.org/dpabi) was performed on each local site. First, the initial 10 volumes were discarded to eliminate magnetic saturation effects. Secondly, the sliding time correction, motion realignment and two‐step spatial normalization to MNI space, linear detrending, and temporal bandpass filtering for 0.01–0.1 Hz were performed in succession. Finally, the filtered image was regressed out the head motion and other nuisances including the global signal, the white matter, and the cerebrospinal fluid to reduce the signal noise [62, 63]. Global signal regression was applied since it was suggested to improve the specificity of positive correlations and can remove specific confounds from the data to facilitate the evaluation of neurophysiological relationships, which makes the results more readily or reliably interpreted [64].

### 5.3 Functional Connectivity Graph

We obtained 1720 regions of interest (ROIs) by fusing multiple atlases, including AAL 116 ROIs [65], Dosenbach 160 ROIs [66], Craddock 200 ROIs [67], Power 264 ROIs [68], and Zalesky 980 ROIs [69]. It was shown that more brain regions can obtain a highly accurate representation but the region interpretability will be sacrificed [67]. To improve classification accuracy, the results of MDD classification were based on 1720 ROIs. To enhance interpretability, the analysis of feature maps and the discriminative connectivity was based on the Power 264 ROIs [68], considering it was widely used and has a clear network partition. Then, time series for the selected ROIs were extracted from the preprocessed fMRI data. Pearson's correlation coefficients between pairs of ROI‐based time series were calculated and normalized to *z*‐scores using Fisher's transformation, resulting in a symmetric connectivity matrix for each session of each subject. The connections were then removed site‐related bias by performing site regression.

The brain connectivity matrix was usually reshaped to a feature vector for further analysis ignoring the spatial information between nodes [15, 62]. To performing graph convolution on connectivity, we must first define a graph structure of brain connectivity for all subjects. In fact, modelling connectivity as an irregular graph could capture the more inherent architecture of the brain network that was related to MDD and help better identify the disorder [70]. We used a *K*‐NN graph G=(V,E) to describe the functional brain connectivity, where its node $v_{i}\in V$ represents a brain ROI, and functional connectivity with all the ROIs serve as node signals $c_{i}\in R^{N},\;i=1,\text{…},N$ and the graph edge $e_{i}\in E$ represents the correlation distance between corresponding node signals. For each node, keep only the edges related to its ten nearest neighbours [29]. We calculated the average functional connectivity of training data to estimate the average functional graph, which will be commonly applied to a graph convolution network for each subject. Note that using the functional metric to represent the graph edges may capture a more accurate brain functional architecture rather than using structural or spatial metrics [29]. Each subject's brain graph was built by taking the ROI as the node, functional connectivity as the edge, and the connections between the current node and all nodes as the features of the current node.

### 5.4 Graph Convolution with Sparse Pooling

To obtain hierarchical embeddings of brain connectome graphs, we proposed a novel GCNSP model. Our model can extend the convolution operation to the brain graph, and obtain hierarchical graph embeddings by learning the local node information, which may contribute to extract underlying site‐invariant and transferable features. Specifically, we built graph convolution layers based on a simple and well‐behaved layer‐wise propagation [28], which achieves better performance than a first‐order approximation of spectral graph convolutions [71]. The layer‐wise propagation rule of the graph convolution network can be represented as follow:

(1) $$H^{\left(l+1\right)}=\sigma \left({\tilde{D}}^{-\frac{1}{2}}\tilde{A}{\tilde{D}}^{-\frac{1}{2}}H^{\left(l\right)}W^{\left(l\right)}\right)$$

Here $W^{\left(l\right)}\in R^{D^{\left(l\right)}\times D^{\left(l+1\right)}}$ is the trainable weight matrix, and $H^{\left(l\right)}\in R^{N\times D^{\left(l\right)}}$ is the activation matrix of the *l^th^* layer. *H* ^(0)^ =  *X*. $\tilde{A}=A+I_{N}$ is the adjacency matrix of graph G with added self‐connections. *I_N_* is the identity matrix denoting the self‐connections. $\tilde{D}$ is a diagonal matrix to normalize the adjacency matrix and ${\tilde{D}}_{ii}=\sum_{i}A_{ij}$. σ(·) represents the activation function, such as the *ReLU*(·).

The output of the last layer of the GCN was regarded as the node embedding of the graph. Given the node embeddings, to deal with the graph‐focus tasks, such as classifying gender labels for the entire brain graph, we must aggregate the features of all nodes to form a graph‐level representation. Moreover, graph representations at different node scales may all contribute to the graph‐focus classification and transfer learning. In order to obtain hierarchical graph representations, we proposed a sparse pooling layer:

(2) $$Z_{G}^{i}=\Theta_{sp}^{i}\;Z_{G}^{i-1}$$

where $\Theta_{sp}^{i}$ is a sparse matrix of the trainable weight of the *i*th sparse pooling layer. $Z_{G}^{i}$ is the output of the *i*th sparse pooling layer. When *i*  =  0, $Z_{G}^{0}=Z$, where *Z* denotes the output of the last GCN layer. Note that the layer can automatically learn the features of sparse nodes and retain only sparse nodes of the graph, which is equivalent to pooling the graph. To take advantage of graph representations of different node scales, we concentrate the output of all sparse pooling layers into the final graph‐level embedding:

(3) $$Z_{G}=\left[Z_{G}^{1},Z_{G}^{2},\text{…},Z_{G}^{n}\right]\;$$

To ensure the sparsity of the weight, an L1 regular term of $\Theta_{sp}^{i}$ is added to the total loss function. Moreover, the sparsity of the weight can be adjusted by changing the corresponding penalty factor.

(4) $$L_{sparse}=\lambda \times \sum_{i}∥\Theta_{sp}^{i}{∥}_{1}$$

where λ is the penalty factor of the L1 regular term of $\Theta_{sp}^{i}$. By adjusting the sparsity of the $\Theta_{sp}^{i}$, we can balance the representation capability and the number of parameters of the sparse pooling, and then select the model with the optimal classification performance.

The advantage of sparse pooling was that it can obtain hierarchical graph embeddings by automatic learning, which can extract a variety of graph features, thus helping to improve classification performance and transfer capability of the GCNSP. Moreover, sparse pooling has fewer trainable parameters, which can significantly improve the generalization of graph convolution, especially for a small dataset.

### 5.5 Network Architecture

Kipf and Welling [28] showed that the best experimental results were obtained when using a two‐layer GCN. Thus, we adopt two‐layer networks with activation function of *ReLU*(·), which can be represented as follows:

(5) $$Z=\;\mathrm{ReLU}\left(\hat{A}\mathrm{ReLU}\left(\hat{A}XW^{\left(0\right)}\right)W^{\left(1\right)}\right)$$

Here 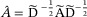 is an intermediate variable just to simplify the formula. *Z* denotes the final output node embeddings. X is the input data. W^(0)^ and W^(1)^ are the trainable weight matrix of input‐to‐hidden and hidden‐to‐output layers, respectively. Based on the node embeddings *Z*, we calculated the graph‐level embeddings Z_*G*_ using the proposed sparse pooling. Then the prediction of the graph label could be computed as:

(6) $$Y_{pred}=\mathrm{softmax}\left(\mathrm{dense}\left(Z_{G}\right)\right)$$

Where, Z_*G*_ is the representation of the entire graph, dense denotes the fully connected output layers. The softmax activation function is defined as $\mathrm{softmax}\;\left(x_{i}\right)=\frac{1}{C}\exp\left(x_{i}\right)$ with $C=\sum_{i}\exp\left(x_{i}\right)$. For supervised graph‐level classification, we evaluate the cross‐entropy error over graph labels as the loss function of prediction. Moreover, to ensure the sparsity of the weight, an L1 regular term of trainable weights in sparse pooling is added to the total loss function:

(7) $$L_{total}=L_{class}+L_{sparse}=-\sum_{i}Y_{pred}\ln\left(Y_{true}\right)+\lambda \times \sum_{i}∥\Theta_{sp}^{i}{∥}_{1}$$

where $L_{class}$ denotes the loss of cross‐entropy between predicted and real gender label, and $L_{sparse}$ denotes the loss of L1 regular term of trainable weights in sparse pooling. Y_*pred*_ denotes the prediction of the graph label, and Y_*true*_ denotes the true graph label. Note that cross‐entropy information has been demonstrated to be an effective loss metric for classification problems.

### 5.6 Multi‐Site Classification

To investigate whether multi‐site transfer learning can improve the classification accuracy on a single‐site, we performed leave‐site‐out transfer learning classification on multi‐site connectome graphs using the proposed GCNSP model. First, we selected a site as the target dataset and aggregated the rest sites as the source dataset. Secondly, we pre‐trained the GCNSP model on the source dataset and then transferred the model to the target dataset to improve classification accuracy. Such transfer learning of a model between the source and target datasets was accomplished with fine‐tuning techniques. The first step of fine‐tuning was to use data from the source dataset for initial training of the model, then save the model parameters. Afterward, 90% of the target dataset was utilized to update the model parameters. Finally, the trained model was tested on the remaining 10% of the target dataset. The finally accuracy was obtained by averaging the accuracies of the ten folds. The classification accuracy based on the RFE‐SVM and the non‐transfer GCNSP model was also calculated for comparison to transfer learning. For each target dataset, we calculated the classification accuracy based on 10‐fold cross validation. In order to avoid the sample size was too small, we selected the sites with more than 20 subjects in either group as the target datasets (obtaining 17 sites; see Figure S4). The Recursive Feature Elimination (RFE) was a feature selection technique that works by recursively removing features from a dataset until the desired number of features was reached. It starts by training a model on all features, ranking them by importance (here we use the *t*‐score of a two‐sample *t*‐test between patients and healthy controls, features with a higher *t*‐score indicates greater importance), and then eliminating the least important ones. This process repeats on the remaining features until a specified number of features is retained (0.01% of the original feature dimension). The procedure aimed to improve model performance and reduce overfitting.

To further show the performance of our GCNSP model, we also performed the following experiments. In multi‐site pooling classification, 10‐fold cross validation was performed on the whole dataset including multiple imaging sites. In leave‐site‐out test classification, a sample of a given site was left for prediction and a sample of the remaining sites was used for model training. As such transfer test classification did not apply the site‐specific feature from the prediction dataset, which reflects the cross‐site generalization of the model. The models of GCNSP, GCN, RFE‐LR, RFE‐SVM and RFE‐LDA were applied to classification for comparison.

### 5.7 Feature Map Visualization

To identify the brain activity patterns learned by the GCNSP model, we visualized the average feature map across subjects for each channel for each network layer (see Figure S6). Note that the feature map represents the output of the corresponding network layer. Moreover, we calculated the overlap ratio between feature maps and functional networks. The correspondence of brain regions and functional networks were determined based on graph analysis [68]. Finally, we calculated the percentage of feature maps associated with each functional network and corrected for the node number of the network.

### 5.8 Class Activation Map

To reconstruct the discriminative functional connectivity for classification, we calculated the class activation maps [72]. Consider the *k* ^th^ feature map *f_k_* (*x*) in the last layer at connectivity x, and $w_{k}^{c}$ the training weights of the last fully connections layer, the class scores (the input to the softmax), *S_c_* is given by the following equivalent:

(8) $$S_{c}=\sum_{k}w_{k}^{c}\;\;\sum_{x}f_{k}\left(x\right)=\sum_{x}\sum_{k}w_{k}^{c}\times f_{k}\left(x\right)\;$$

Noted that the second equivalent is obtained by swapping the summation operators. The class activation map *M_c_* is then defined as $\sum_{k}w_{k}^{c}\times f_{k}\left(x\right)$, which reflects the contribution of each connectivity to graph classification, i.e., *M_c_* is the discriminative activation scores of the brain functional connectivity for graph classification.

### 5.9 Statistical Differences Between Groups

To evaluate the inter‐group differences of feature maps (or functional connectivity), we performed two sample *t*‐test on each element of the feature map (or each functional connection), and estimated the distribution probability of *t* values using kernel density estimation [73]. It was known that the larger absolute value of *t*, the greater the inter‐group differences. Therefore, it was easy to reason that the larger the value at both ends of the distribution probability curve, the greater the difference between the groups.

### 5.10 Hyperparameters Setting

Because hyperparameters optimization requires huge calculations and a validation set needs to be divided, which will result in fewer training samples, we have not comprehensively optimized the hyperparameters of the model. For RFE‐LDA, RFE‐LR, and RFE‐SVM models, we used the default parameters in MATLAB, and the ratio of the selected features was set to optimized 0.0001. For GCN and GCNSP, we set the hyperparameters to commonly used parameters based on previous research [29]: learning rate = 0.0001, hidden size = 64, epoch = 100, L2 regularization = 0.0001, pooling ratio = 0.5, dropout ratio = 0.5, and penalty factor on the sparse pooling = 0.0005. The batch size was set to 32 for multi‐site large sample training, while it was set to 16 for single‐site small sample training. Note that other hyperparameters were used the same for multi‐site pooling classification and multi‐site transfer learning classification. For feature maps and discriminative connectivity analysis, in order to facilitate interpretability analysis, the hidden size was set to 16. For multi‐site transfer learning classification, because the number of samples in each site was different, we also evaluated the classification results by selecting the optimal epoch for each site.

## Author Contributions

J.S., J.Q., H.S., L.Z., L.L., and D.H. designed the project. J.S., J.Q., L.Z., and D.H. conceived and performed transfer learning studies. J.Q., J.S., H.S., and L.L. conceived and performed graph convolution network studies. J.Q., J.S., L.Z., B.L., J.L., X.S., W.P., T.B., H.W., H.Y., K.W., and L.L. collected the fMRI data and performed data preprocessing. J.Q. and J.S. wrote the manuscript and all the authors provided feedback on the manuscript. The revision of the manuscript is mainly accomplished by J.S.

## Conflicts of Interest

The authors declare no conflicts of interest.

## Supporting information




**Supporting File**: advs74016‐sup‐0001‐SuppMat.docx.

## Data Availability

The functional connectivity data of all subjects is available on the website of https://pan.baidu.com/s/112X8Ogs4oDJnU8lxcb3pWg, with the extraction code of x3sv. 1720‐ROI timecourses of subjects in Site 26 to Site 33 are available on the website of https://pan.baidu.com/s/189USxgWQ‐qU1i7TQXq5u6A, with the extraction code of kx4c. The unprocessed raw data will be provided upon request. For the subjects from the REST‐Meta‐MDD project, there is a formal process to access unprocessed raw data that will be reviewed by the consortium investigators. The codes for multi‐site pooling classification via RFE‐LDA, RFE‐LR, RFE‐SVM, GCN, and GCNSP, as well as for multi‐site transfer learning classification via GCNSP are both available on https://github.com/Qin‐J/Multi‐site‐transfer‐classification‐of‐major‐depressive‐disorder. The data that support the findings of this study are openly available in Rest‐meta‐MDD at [DOI:http://rfmri.org/REST-meta-MDD], reference number 61.
