# Supplementary material for: Multi‐Site Transfer Classification of Major Depressive Disorder: An fMRI Study in 3335 Subjects
Source: Adv Sci (Weinh). 2026 Jan 29;13(19):e02817. doi: 10.1002/advs.202502817 (PMC13045371; doi:10.1002/advs.202502817)
Supplement: Supplementary file 1 — Supporting File: advs74016‐sup‐0001‐SuppMat.docx. [file ADVS-13-e02817-s001.docx]

Table S1. Subject number and data acquisition parameters for each consortium site

| Sites index | Sites(cohorts) | Subject Number | | Scanner | Receive  (coil) | TR (ms) | TE  (ms) | Flip Angle (∘) | Thickness  /gap (mm) | Slice number | Time points | Voxel  size | FOV (mm^2^) |
| --- | --- | --- | --- | --- | --- | --- | --- | --- | --- | --- | --- | --- | --- |
|  |  | MDD | NC |  |  |  |  |  |  |  |  |  |  |
| 1 | National Clinical Research Center for Mental Disorders (Peking University Sixth Hospital) & Key Laboratory of Mental Health, Ministry of Health (Peking University) | 74 | 74 | Siemens Tim Trio 3.0T | 32 channel | 2000 | 30 | 90 | 4.0/0.8 | 30 | 210 | 3.28×  3.28×  4.80 | 210×210 |
| 2 | Department of Clinical Psychology, Suzhou Suzhou Psychiatric Hospital, The Affiliated Guangji Hospital of Soochow University | 30 | 30 | Philips Achieva 3.0T | 8  channel | 2000 | 30 | 90 | \| 4.0/0 \| \| --- \| | 37 | 200 | 1.67×  1.67×  4.00 | 240×240 |
| 3 | The Second Xiangya Hospital of Central South University | 27 | 37 | Siemens Magnetom Symphony Scanner 1.5 T | 16  channel | 2000 | 40 | 90 | 5.0/1.25 | 26 | 150 | 3.75×  3.75×  6.25 | 240×240 |
| 4 | The Second Xiangya Hospital of Central South University | 24 | 24 | Siemens Skyra 3.0T | 32  channel | 2500 | 25 | 90 | 3.5/0 | 39 | 200 | 3.75×  3.75×  3.50 | 240×240 |
| 5 | Department of Psychiatry, Shanghai Jiao Tong University School of Medicine | 13 | 11 | GE Signa 3.0T | 32  channel | 3000 | 30 | 90 | 5.0/0 | 22 | 100 | 3.75×  3.75×  5.00 | 240×240 |
| 6 | Department of Psychiatry, Shanghai Jiao Tong University School of Medicine | 15 | 15 | Siemens Tim Trio 3.0T | 32  channel | 2000 | 30 | 70 | 4/0 | 33 | 180 | 3.59×  3.59×  4.00 | 230×230 |
| 7 | Sir Run Run Shaw Hospital, Zhejiang University School of Medicine | 38 | 49 | GE discovery MR750  3.0T | 8  channel | 2000 | 30 | 90 | 3.2/0 | 37 | 184 | 2.29×  2.29×  3.20 | 220×220 |
| 8 | Department of Psychiatry, First Affiliated Hospital, China Medical University | 75 | 75 | GE Signa 3.0T | 8  channel | 2000 | 30 | 90 | 3.0/0 | 35 | 200 | 3.75×  3.75×  3.00 | 240×240 |
| 9 | The First Affiliated Hospital of Jinan University | 50 | 50 | GE Discovery MR750 3.0T | 8  channel | 2000 | 25 | 90 | 3.0/1.0 | 35 | 200 | 3.75×  3.75×  4.00 | 240×240 |
| 10 | First Hospital of Shanxi Medical University | 50 | 33 | Siemens Tim Trio 3.0T | 32  channel | 2000 | 30 | 90 | 3.0/1.52 | 32 | 212 | 3.75×  3.75×  4.52 | 240×240 |
| 11 | Department of Psychiatry, The First Affiliated Hospital of Chongqing Medical University | 32 | 29 | GE Signa 3.0T | 8  channel | 2000 | 30 | 90 | 5/0 | 33 | 200 | 3.75×  3.75×  5.00 | 240×240 |
| 12 | Department of Psychiatry, The First Affiliated Hospital of Chongqing Medical University | 32 | 6 | GE Signa 3.0T | 8  channel | 2000 | 30 | 90 | 5/0 | 33 | 240 | 3.75×  3.75×  4.00 | 240×240 |
| 13 | The First Affiliated Hospital of Xi’an Jiaotong University, Xi’an Central Hospital | 25 | 17 | GE Excite 1.5T | 16  channel | 2500 | 35 | 90 | 4/0 | 36 | 150 | 4.00×  4.00×  4.00 | 256×256 |
| 14 | The Second Xiangya Hospital of Central South University | 64 | 32 | Siemens Tim Trio 3.0T | 32  channel | 2500 | 25 | 90 | 3.5/0 | 39 | 200 | 3.75×  3.75×  3.50 | 240×240 |
| 15 | Department of Psychosomatics and Psychiatry, Zhongda Hospital, School of Medicine, Southeast University | 50 | 50 | Siemens Verio 3.0T | 12  channel | 2000 | 25 | 90 | 4/0 | 36 | 240 | 3.75×  3.75×  4.00 | 240×240 |
| 16 | Huaxi MR Research Center, West China Hospital of Sichuan University | 31 | 31 | GE Signa 3.0T | 8  channel | 2000 | 30 | 90 | 5.0/0 | 30 | 200 | 3.75×  3.75×  5.00 | 240×240 |
| 17 | Department of Psychiatry, The First Affiliated Hospital of Chongqing Medical University | 47 | 44 | GE Signa 3.0T | 8  channel | 2000 | 40 | 90 | 4.0/0 | 33 | 240 | 3.75×  3.75×  4.00 | 240×240 |
| 18 | Department of Radiology, The First Affiliated Hospital, College of Medicine, Zhejiang University | 21 | 20 | Philips Achieva 3.0T | 8channel  SENSE  Head coil | 2000 | 35 | 90 | 5.0/1.0 | 24 | 200 | 1.67×  1.67×  6.00 | 240×240 |
| 19 | Anhui Medical University | 51 | 36 | GE Signa 3.0T | 8  channel | 2000 | 22.5 | 30 | 4.0/0.6 | 33 | 240 | 3.44×  3.44×  4.60 | 220×220 |
| 20 | Faculty of Psychology, Southwest University | 282 | 251 | Siemens Tim Trio 3.0T | 12  channel | 2000 | 30 | 90 | 3.0/1.0 | 32 | 242 | 3.44×  3.44×  4.00 | 220×220 |
| 21 | Beijing Anding Hospital, Capital Medical University | 86 | 70 | Siemens Tim Trio 3.0T | 32  channel | 2000 | 30 | 90 | 3.5/0.7 | 33 | 240 | 3.12×  3.12×  4.20 | 200×200 |
| 22 | The Institute of Mental Health, Second Xiangya Hospital of Central South University | 30 | 20 | Philips Gyroscan Achieva 3.0T | 32  channel | 2000 | 30 | 90 | 4.0/0 | 36 | 250 | 1.67×  1.67×  4.00 | 240×240 |
| 23 | Mental Health Center, West China Hospital, Sichuan University | 32 | 30 | Philips Achieva 3.0T TX | 8  channel | 2000 | 30 | 90 | 4.0/0 | 38 | 240 | 3.75×  3.75×  6.50 | 240×240 |
| 24 | First Affiliated Hospital of Kunming Medical University | 32 | 31 | GE Signa 1.5T | 8  channel | 2000 | 40 | 90 | 5.0/1.0 | 24 | 160 | 3.75×  3.75×  6.00 | 240×240 |
| 25 | Department of Neurology, Affiliated ZhongDa Hospital of Southeast University | 89 | 63 | Siemens Verio 3.0T | 12  channel  Head coil | 2000 | 25 | 90 | 4.0/0 | 36 | 240 | 3.75×  3.75×  4.00 | 240×240 |
| 26 | The First Affiliated Hospital of Anhui Medical University | 106 | 47 | GE Signa 3.0T | 8  channel | 2000 | 22.5 | 30 | 4.0/0.6 | 33 | 240 | 3.44×  3.44×  4.60 | 220×220 |
| 27 | The First Affiliated Hospital of China Medical University | 24 | 29 | GE Signa 1.5T | 8  channel | 2000 | 50 | 90 | 5.0/1.5 | 20 | 240 | 3.75×  3.75×  6.50 | 240×240 |
| 28 | Xijing Hospital, Fourth Military Medical University | 62 | 67 | Siemens Tim Trio 3.0T | 12  channel | 2000 | 30 | 90 | 4.0/0.6 | 33 | 240 | 3.44×  3.44×  4.60 | 220×220 |
| 29 | Xijing Hospital, Fourth Military Medical University | 43 | 59 | GE Signa 3.0T | 8  channel | 2000 | 22.5 | 30 | 4.0/0.6 | 33 | 240 | 3.44×  3.44×  4.60 | 220×220 |
| 30 | The Second Xiangya Hospital of Central South University | 158 | 117 | Siemens Magnetom Symphony 1.5T | 8  channel | 2000 | 40 | 90 | 5.0/1.25 | 26 | 150 | 3.75×  3.75×  6.25 | 240×240 |
| 31 | The Second Xiangya Hospital of Central South University | 20 | 43 | Philips Gyroscan Achieva 3.0T | 12  channel | 2000 | 30 | 90 | 4.0/0 | 36 | 250 | 3.75×  3.75×  4.00 | 240×240 |
| 32 | The Second Xiangya Hospital of Central South University | 42 | 51 | GE Signa 1.5T | 8  channel | 2000 | 40 | 90 | 5.0/1.0 | 20 | 150 | 3.75×  3.75×  6.00 | 240×240 |
| 33 | Department of Radiology, The First Affiliated Hospital, College of Medicine, Zhejiang University | 13 | 26 | GE Discovery MR750 3.0T | 32  channel | 2000 | 30 | 90 | 3.2/0 | 37 | 250 | 3.44×  3.44×  4.60 | 220×220 |

**Abbreviations: MDD, major depressive disorder; NC, normal control.**


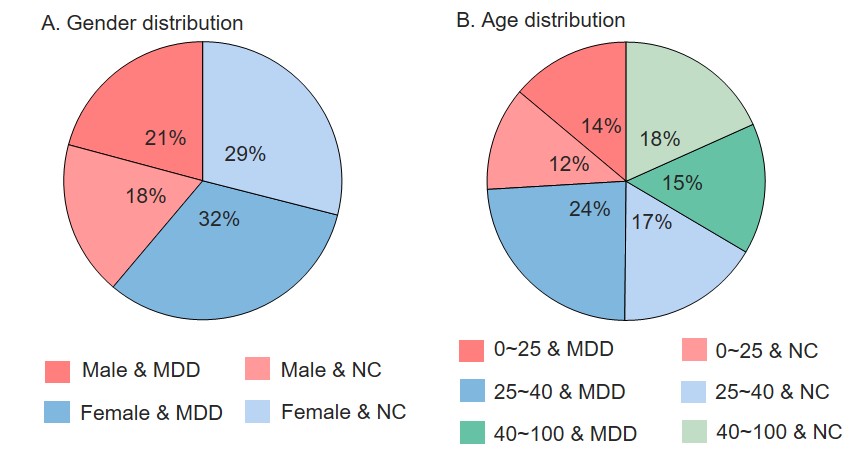
 Figure S1. Pie chart of gender distribution (A) and age distribution (B). MDD, major depressive disorder; NC, normal control.

***
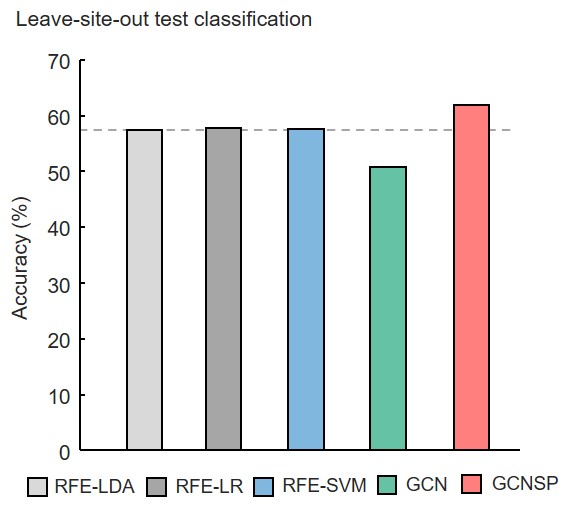
***

Figure S2. The accuracy of leave-site-out test classification. RFE, recursive feature elimination; LDA, linear discriminant analysis; LR, logistic regression; SVM, support vector machine; GCN, graph convolution network; GCNSP, graph convolution network with sparse pooling.


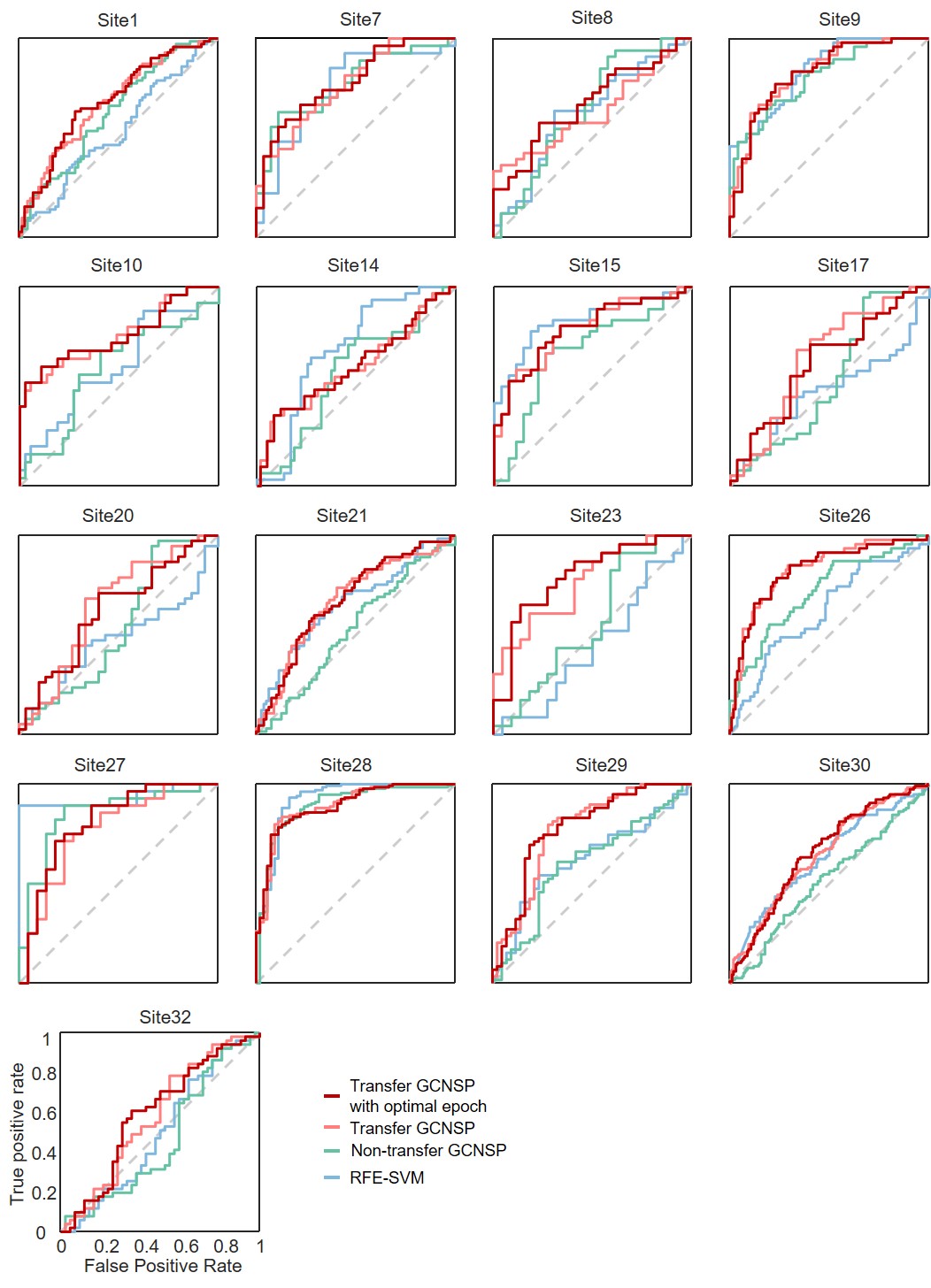


Figure S3. The receiver operating characteristic curve of RFE-SVM, non-transfer GCNSP, and transfer GCNSP on each site. Note that in order to avoid the sample size is too small, we selected the 17 sites with more than 20 subjects in either group as the target dataset.


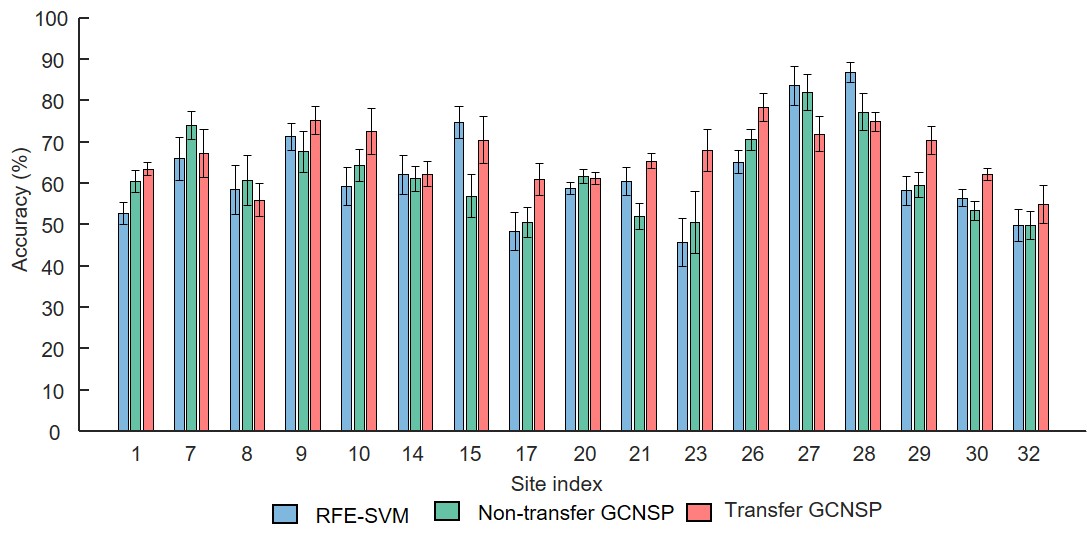


Figure S4. The classification accuracy for each site using RFE-SVM, the non-transfer and transfer learning of GCNSP. Note that the classification accuracy of each site was evaluated based on the ten-fold cross validation. In order to avoid the sample size is too small, we selected the 17 sites with more than 20 subjects in either group as the target dataset.

Table S2. Subject information on multi-sites of healthy datasets for gender classification.

| datasets | subjects (M/F) | scans (M/F) | age (M/F) |
| --- | --- | --- | --- |
| Cam-Can | 319/328 | 319/328 | 18-87/18-88 |
| CoRR | 630/612 | 1799/1837 | 6-83/6-88 |
| FCP | 533/627 | 594/694 | 9-74/8-78 |
| GSP | 665/904 | 1131/1574 | 19-35/19-35 |
| HCP | 500/596 | 1944/2289 | 22-35/22-35 |
| MBB | 189/129 | 555/440 | 20-75/20-75 |
| NKI-RS | 232/363 | 246/399 | 7-82/8-85 |
| OASIS-3 | 310/451 | 1054/1608 | 43-89/43-95 |
| SALD | 187/305 | 187/305 | 20-80/19-78 |
| SLIM | 262/332 | 441/568 | 17-26/17-27 |
| all sites | 3827/4647 | 8270/10042 | 6-89/6-95 |

***
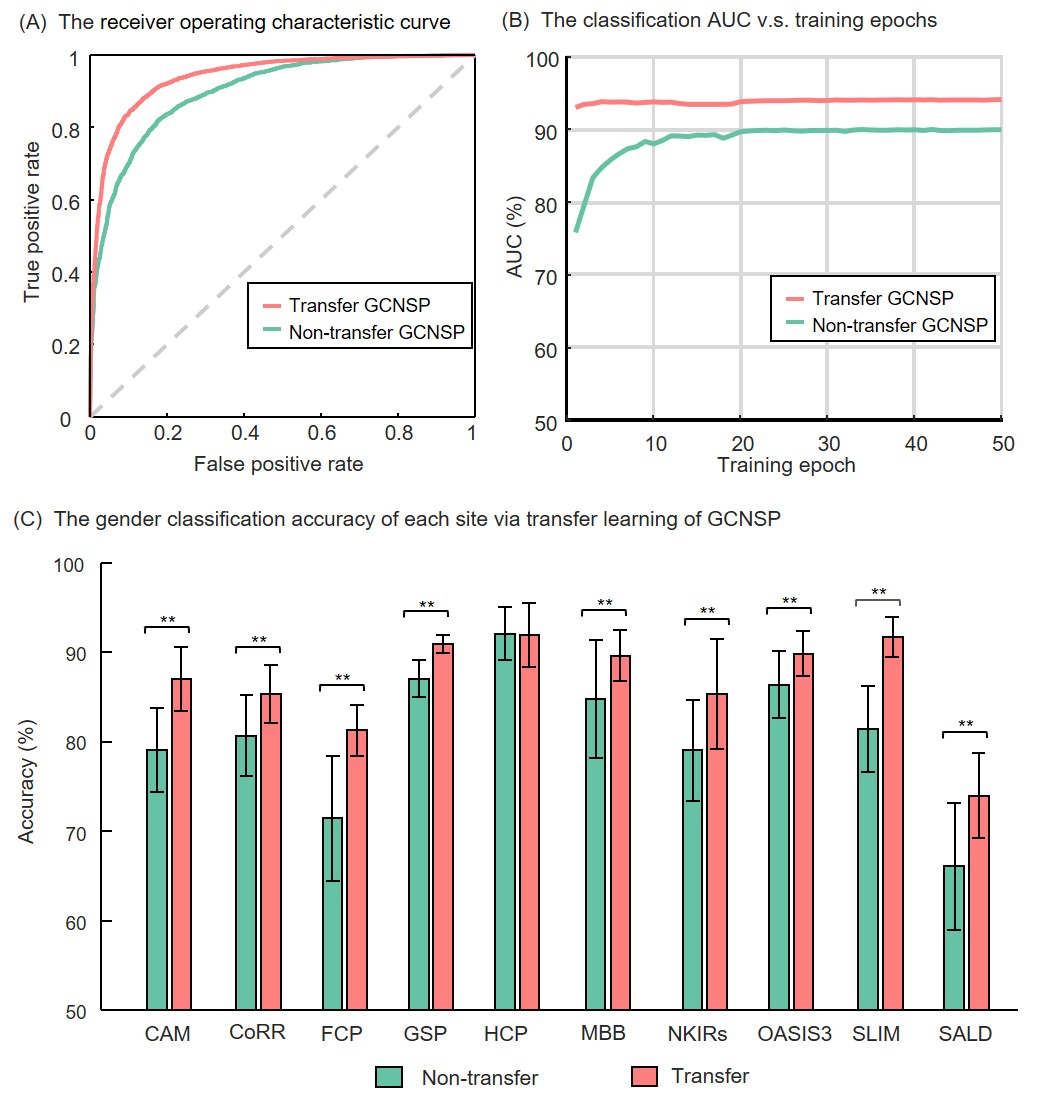
***

Figure S5. The classification accuracy of gender based on the non-transfer and transfer learning of GCNSP. (A) The average receiver operating characteristic curve of RFE-SVM, non-transfer GCNSP, and transfer GCNSP on each site. (B) The average classification AUC of multiple models changes with different training epochs. (C) The classification accuracy for each site using RFE-SVM and transfer learning of GCNSP. Note that the classification accuracy of each site was evaluated based on the ten-fold cross validation. We applied 10 publicly released datasets, including the Cambridge Centre for Ageing and Neuroscience (Cam-CAN) [^1^](#_ENREF_1), the Consortium for Reliability and Reproducibility (CoRR) [^2^](#_ENREF_2), the 1000 Functional Connectomes Project (FCP) [^3^](#_ENREF_3), the Brain Genomics Superstruct Project (GSP) [^4^](#_ENREF_4), the Human Connectome Project (HCP) [^5^](#_ENREF_5), the Mind-Brain-Body Dataset (MBB) [^6^](#_ENREF_6), the enhanced Nathan Kline Institute-Rockland Sample (NKI-RS) [^7^](#_ENREF_7), the latest release in the Open Access Series of Imaging Studies (OASIS-3) [^8^](#_ENREF_8)^,^ [^9^](#_ENREF_9), the Southwest University Longitudinal Imaging Multimodal (SLIM) [^10^](#_ENREF_10), and the Southwest University Adult Lifespan Dataset (SALD) [^11^](#_ENREF_11). Some repeated subjects (18 female subjects from the CoRR database were repeated in the NKI database; 156 female and 134 male subjects were repeated in the SLIM database), subjects missing gender information, or data that were not subjected to the data preprocessing pipeline due to poor imaging quality were removed. Therefore, the whole dataset contains a total of 1,7696 scans from 8166 healthy subjects. For transfer learning, a single site is used as the target dataset, and the remaining sites are used as the source dataset. Excluding HCP, the transfer model significantly improved the classification accuracy of each site compared with the baseline model. ** represent *P* < 0.05 in Wilcoxon rank-sum test.


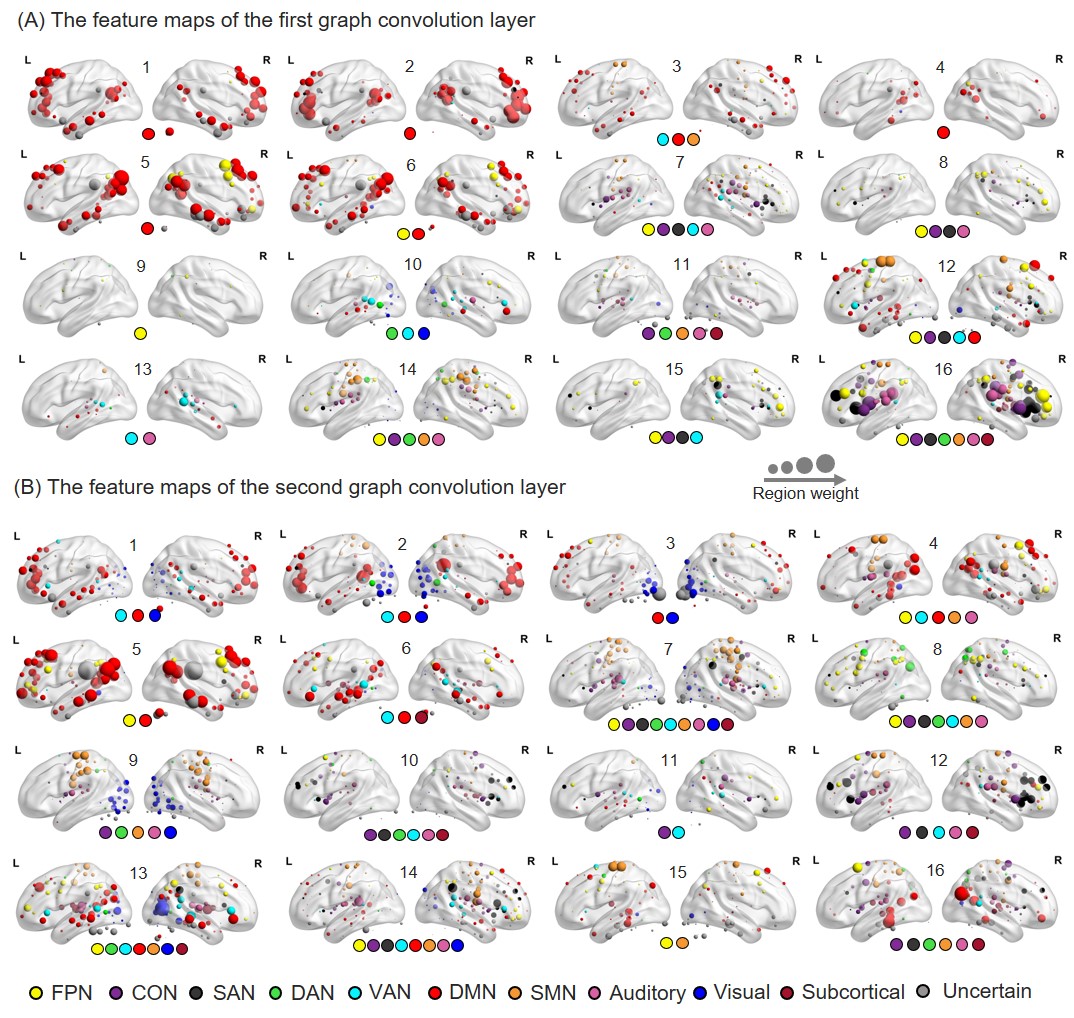


Figure S6. The feature maps of the first (A) and second (B) graph convolution layers. The sphere represents the region of interest, and the size denotes the weight contributed to classification and the colour denotes the brain functional network. The number at the top of the brain map indicates the channels, and the coloured sphere at the bottom represents the activated network.

Table S3. Classification accuracy (%) and site-specific factors

| **Site** | 1 | 7 | 8 | 9 | 10 | 14 | 15 | 17 | 20 | 21 | 23 | 26 | 27 | 28 | 29 | 30 | 32 |
| --- | --- | --- | --- | --- | --- | --- | --- | --- | --- | --- | --- | --- | --- | --- | --- | --- | --- |
| **Samples** | 146 | 72 | 116 | 96 | 71 | 93 | 67 | 82 | 479 | 144 | 45 | 153 | 50 | 121 | 102 | 275 | 93 |
| **Scanner** | 1 | 2 | 3 | 2 | 1 | 1 | 4 | 3 | 1 | 1 | 5 | 3 | 6 | 1 | 3 | 7 | 6 |
| **MFI** | 3T | 3T | 3T | 3T | 3T | 3T | 3T | 3T | 3T | 3T | 3T | 3T | 1.5T | 3T | 3T | 1.5T | 1.5T |
| **Age** | 31.7 | 42.6 | 28.6 | 28.1 | 33.0 | 30.5 | 46.9 | 21.0 | 38.7 | 34.7 | 18.6 | 39.4 | 31.8 | 34.9 | 35.2 | 34.3 | 23.7 |
| **HAMD** | 24.9 | 22.2 | 22.9 | N/A | 20.7 | 21.3 | 27.3 | 20.8 | 20.9 | 14.4 | 18.6 | N/A | 26.4 | N/A | 23.4 | 22.0 | N/A |
| **Acc%** | 60.57 | 74.00 | 60.67 | 67.67 | 64.29 | 61.11 | 56.90 | 50.48 | 61.91 | 52.71 | 50.50 | 70.54 | 82.00 | 77.26 | 60.64 | 53.80 | 48.67 |

**Scanner**: 1- Siemens Tim Trio 3.0T, 2- GE discovery MR750 3.0T, 3- GE Signa 3.0T, 4- Siemens Verio 3.0T, 5- Philips Achieva 3.0T TX, 6- GE Signa 1.5T, 7- Siemens Magnetom Symphony 1.5T.

**MFI**: magnetic field intensity.

**Age**: Average age of patients with MDD.

**HAMD**: The average Hamilton Rating Scale for Depression of patients in each site (if available).

**Potential factors for poor classification performance**.

Correlation analysis between site-specific factors, i.e., sample size, scanner types and patient demographics, have been done to further unveil the potential source for poor classification performance. We first conduct Pearson’s correlation analysis between sample size and classification accuracy. The resulting *r* = -0.0922 and *p* = 0.7249, which indicates that classification accuracy is not correlated with sample size. As for patient demographics, we utilize the average age and HAMD to represent the overall conditions of patients in each site. Pearson’s correlation analysis between site-specific patient demographics and classification accuracy is done. For age of patient and classification accuracy, the resulting *r* = 0.4239 and *p* = 0.0899. For HAMD and classification accuracy, the resulting *r* = 0.4911 and *p* = 0.0883. Both of them are at the margin of significance.

**Sample exclusion.**

1) Site 25 was excluded since it mainly contained late onset depression (most with age > 60) and remitted patients; 2) subjects without information on sex, age and education were excluded; 3) Subjects with bad imaging data and bad spatial normalization (by visual inspection) were excluded; 4) subjects with age less than 18 or more than 65 were excluded; 5) subjects with bad coverage (<90% of the group mask) or excessive head motion (mean FD > 0.2mm) were excluded; 6) to further remove subjects with distortions that not screened by visual inspection, we excluded subjects with spatial correlation < 0.6 (a threshold defined by mean - 2SD) between each participant’s ReHo map and the group mean ReHo map.

**Reference:**

1. Shafto, M.A. et al. The Cambridge Centre for Ageing and Neuroscience (Cam-CAN) study protocol: a cross-sectional, lifespan, multidisciplinary examination of healthy cognitive ageing. *BMC Neurology* **14**, 204 (2014).

2. Zuo, X.-N. et al. An open science resource for establishing reliability and reproducibility in functional connectomics. *Scientific data* **1**, 140049 (2014).

3. Mennes, M., Biswal, B.B., Castellanos, F.X. & Milham, M.P. Making data sharing work: the FCP/INDI experience. *Neuroimage* **82**, 683-691 (2013).

4. Holmes, A.J. et al. Brain Genomics Superstruct Project initial data release with structural, functional, and behavioral measures. *Scientific data* **2**, 150031 (2015).

5. Smith, S.M. et al. Resting-state fMRI in the human connectome project. *Neuroimage* **80**, 144-168 (2013).

6. Babayan, A. et al. A mind-brain-body dataset of MRI, EEG, cognition, emotion, and peripheral physiology in young and old adults. *Scientific data* **6** (2019).

7. Nooner, K. et al. The NKI-Rockland Sample: A Model for Accelerating the Pace of Discovery Science in Psychiatry. *Frontiers in Neuroscience* **6** (2012).

8. Marcus, D.S. et al. Open Access Series of Imaging Studies (OASIS): cross-sectional MRI data in young, middle aged, nondemented, and demented older adults. *Journal of Cognitive Neuroscience* **19**, 1498-1507 (2007).

9. Marcus, D.S., Fotenos, A.F., Csernansky, J.G., Morris, J.C. & Buckner, R.L. Open access series of imaging studies: longitudinal MRI data in nondemented and demented older adults. *Journal of Cognitive Neuroscience* **22**, 2677-2684 (2010).

10. Liu, W. et al. Longitudinal test-retest neuroimaging data from healthy young adults in southwest China. *Scientific data* **4**, 170017 (2017).

11. Wei, D. et al. Structural and functional brain scans from the cross-sectional Southwest University adult lifespan dataset. *Scientific data* **5** (2018).
